# Association of tumor location with anxiety and depression in childhood brain cancer survivors: a systematic review and meta-analysis

**DOI:** 10.1186/s13034-023-00665-0

**Published:** 2023-10-27

**Authors:** Márton Szabados, Erika Kolumbán, Gergely Agócs, Szilvia Kiss-Dala, Marie Anne Engh, Márk Hernádfői, Kata Takács, Eszter Tuboly, Andrea Párniczky, Péter Hegyi, Miklós Garami

**Affiliations:** 1 Pediatric Center, Semmelweis University, 7-9 Tűzoltó Street, Budapest, 1094 Hungary; 2 Centre for Translational Medicine, Semmelweis University, Budapest, Hungary; 3 Institute for Translational Medicine, Medical School, University of Pécs, Pécs, Hungary; 4 Bethesda Children’s Hospital, Budapest, Hungary; 5 András Pető Faculty, Semmelweis University, Budapest, Hungary; 6 Heim Pál National Pediatric Institute, Budapest, Hungary; 7 Institute of Pancreatic Diseases, Semmelweis University, Budapest, Hungary; 8 Department of Biophysics and Radiation Biology, Semmelweis University, Budapest, Hungary; 9 Hungarian Pediatric Oncology Network, Budapest, Hungary

**Keywords:** Pediatric, Brain tumor, Tumor location, Depression, Anxiety

## Abstract

**Objective:**

This study aimed to evaluate the association between the location (supratentorial or infratentorial) of brain tumors and the development of depression and anxiety in childhood cancer survivors. Understanding the risk factors for the development of depression and anxiety disordersin these patients is crucial for early diagnosis and successful treatment.

**Methods:**

The meta-analysis included articles that listed patients diagnosed with an intracranial tumor before the age of 18 years, provided the location of the tumor, had exact data on the prevalence of anxiety and depression, or measured these disorders using different assessment tools. The search was conducted in five different databases (MEDLINE, Embase, Web of Science, Scopus, and Cochrane Library). Risk of bias was assessed using QUIPS-2. Outcome measures used were prevalences and standardized means.

**Results:**

The analysis included 42 eligible articles with a total number of 1071 patients. Relevant articles were cohort studies, cross-sectional studies, and case series. Based on the available data infratentorial brain tumor survivors had significantly higher scores on various assessment tools measuring anxiety (MRAW (raw mean scores): 36.24 [CI (confidence interval): 28.81–43.67]; versus MRAW: 23.21 (CI 0.91–45.51); p = 0.02, and depression (MRAW: 27.57 (CI 14.35–40.78) versus MRAW: 13.84 (CI 11.43–16.26); p < 0.01.

**Conclusion:**

Childhood infratentorial cancer survivors have more impairments in terms of depression and anxiety; these children and adults should be monitored more frequently and may require closer follow-up on their mental health. The main limitation of our study originates from the lack of data on follow-up times used by different studies.

**Supplementary Information:**

The online version contains supplementary material available at 10.1186/s13034-023-00665-0.

## Introduction

While the majority of children who survive cancer are able to deal with the stressors associated with their illness without any psychological issues and even benefit (e.g., willingness to express emotions to others, appreciation of life) from the experience [1, 2], many children and the majority of adult survivors of pediatric brain cancer may show various symptoms of psychological distress, such as anxiety and depression [3, 4].

This problem has become important over the past few decades because the survival rate of pediatric brain tumors has significantly improved, with overall 5-year survival rates above 70% in high-income countries, as treatment modalities, including surgical resection, irradiation, chemotherapy, and targeted therapies, have improved and become more effective [5, 6]. The focus has shifted more to understanding the long-term consequences as well as the remaining function after treatment [7].

Early detection and treatment of emotional disorders are of utmost importance. It becomes simpler to reduce the severity and duration of symptoms, avoid relapse, and improve overall functioning if diagnosed at an early stage. For instance, conditions such as anxiety disorders, depressive disorders, and post-traumatic stress disorder can significantly impact physical and mental health [8]. However, early intervention can significantly improve the well-being of an individual and help prevent the development of more severe symptoms and complications, such as impaired daily functioning [9, 10].

Understanding the risks associated with the development of affective disorders in brain tumor survivors is crucial for facilitating faster and more efficient diagnosis [11]. Tumor location is considered one possible risk factor [12]. As these diseases are complex conditions, the exact brain regions involved in their development may vary from person to person and are likely to result from the interaction of multiple brain regions and other factors, such as genetics, environment, and life experiences. However, research suggests that several key brain regions are involved in affective disorders, including the prefrontal cortex, amygdala, hippocampus, and thalamus [13]. Supra-and infratentorial brain tumors have different etiologies, histologies, and symptoms [28]. It is, however, still unknown how these locations impact the development of depression and anxiety.

The aim of this meta-analysis was to determine the relationship between the location of the brain tumor and the prevalence of anxiety disorder and major depression, in order to provid professionals with better options for diagnosis and interventions designed to treat psychological disorders at an early stage.

## Methods

The Preferred Reporting Items for Systematic Reviews and Meta-Analyses (PRISMA) statement was used to report the findings of our study, while we followed the Cochrane Handbook for methodological guidance [14]. The protocol was registered on PROSPERO (CRD42022370756).

### Search strategy

A systematic search was performed in five scientific databases: Medline (via PubMed), Embase, Cochrane Central Register of Controlled Trials (CENTRAL), Scopus, and Web of Science. The search date was 22 November 2022. Reference lists were also screened to capture all relevant studies via ‘citationchaser’ [15]. The search key we used included the following domains: pediatrics, brain cancer, location, and affective disorders (Additional file 1).

### Selection and eligibility criteria

After duplicates were removed manually and the reference management software (EndNote X9) was used, a pair of review authors independently screened articles by title, abstract, and full text based on predefined eligibility criteria. Cohen’s kappa was used to assess interrater agreements during selection. Conflicts in selection were resolved by a third author. No language or other restrictions were applied during the search.

The inclusion criteria were the following:

- Patients diagnosed with any type of intracranial malignancies before the age of 18,
- Clear information on the location of the brain tumor in the involved articles (supratentorial or infratentorial and brain tumor types that are specific for one of the main locations),
- A defined diagnosis of affective disorders (based on DSM-IV or DSM-V) or exact data on scores of different questionnaires (HADS, CDI, CDI-2, BSI-18, SCARED, CBCL) measuring depressive and anxiety symptoms.

We excluded case report studies, articles that did not include the specific location of the tumor and studies that only mentioned impairments in different affective aspects without any data.

### Data extraction

Two independent review authors extracted data from eligible studies using a standardized data collection form. All disagreements were resolved by an independent third author. The following data were extracted from each included study: authors, publication year, digital object identifier, study design, study period, number of patients included in each study, number of patients diagnosed with depression and anxiety, mean scores, and standard deviations of the different questionnaires, and follow-up times.

### Risk of bias assessment

The Quality in Prognostic Studies 2 (QUIPS-2) tool was used by two independent review authors. Any disagreements were resolved by a third investigator.

### Statistical analysis

The meta-analyses were performed with R 4.1.2 (R Core Team. 2022. R: A Language and Environment for Statistical Computing. Vienna, Austria: R Foundation for Statistical Computing. https://www.R-project.org/.), using the {meta} package (version: 6.1.0) [16].

The prevalence of depression and anxietyin childhood brain cancer survivors was estimated by pooling the proportions of patients who later developed depression or anxiety.

The severity of depression and anxiety was assessed using several questionnaires. The scores of CBCL (Child Behavior Checklist) questionnaires were analyzed by pooling raw T-score values. For depression and anxiety, only standardized mean values could be pooled, as different questionnaires used different scales. Scores were standardized by transforming the range of the scale of the original questionnaires to a common scale ranging from 0 to 100, showing as raw mean scores (MRAW).

Subgroup analysis was performed based on tumor location.

Random intercept logistic regression models were used for prevalences. Heterogeneity variance (tau squared) was estimated using the maximum-likelihood method. Clopper-Pearson confidence intervals were calculated for each study [17].

In the case of raw means, random-effects models were applied with the inverse variance method. A maximum-likelihood estimator restricted to tau squared was used. Hartung-Knapp adjustments were used due to the low number of studies [18].

Heterogeneity was tested using Cochrane’s Q [N + 3] and Higgins & Thompson’s I squared statistics [19, 20].

## Results

### Results of search and selection

**Fig. 1:**
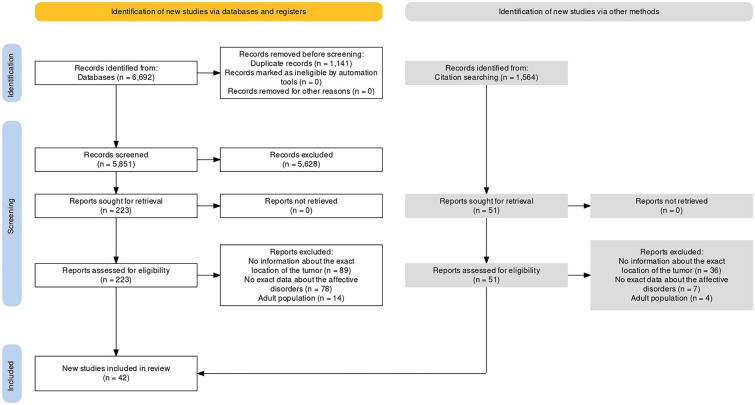
PRISMA flow diagram tool chart illustrating the selection process [21]

After conducting the search, we identified 6692 records and 1564 records through the references. A total of 42 studies were included in the quantitative analysis. The selection process is described in the PRISMA flowchart (Fig. 1).

### Characteristics of studies included

All studies presented and analyzed were cohort (retrospective and prospective), case-series, or cross-sectional (Additional file 2).

### Brain tumor location and the prevalence of depression

**Fig. 2:**
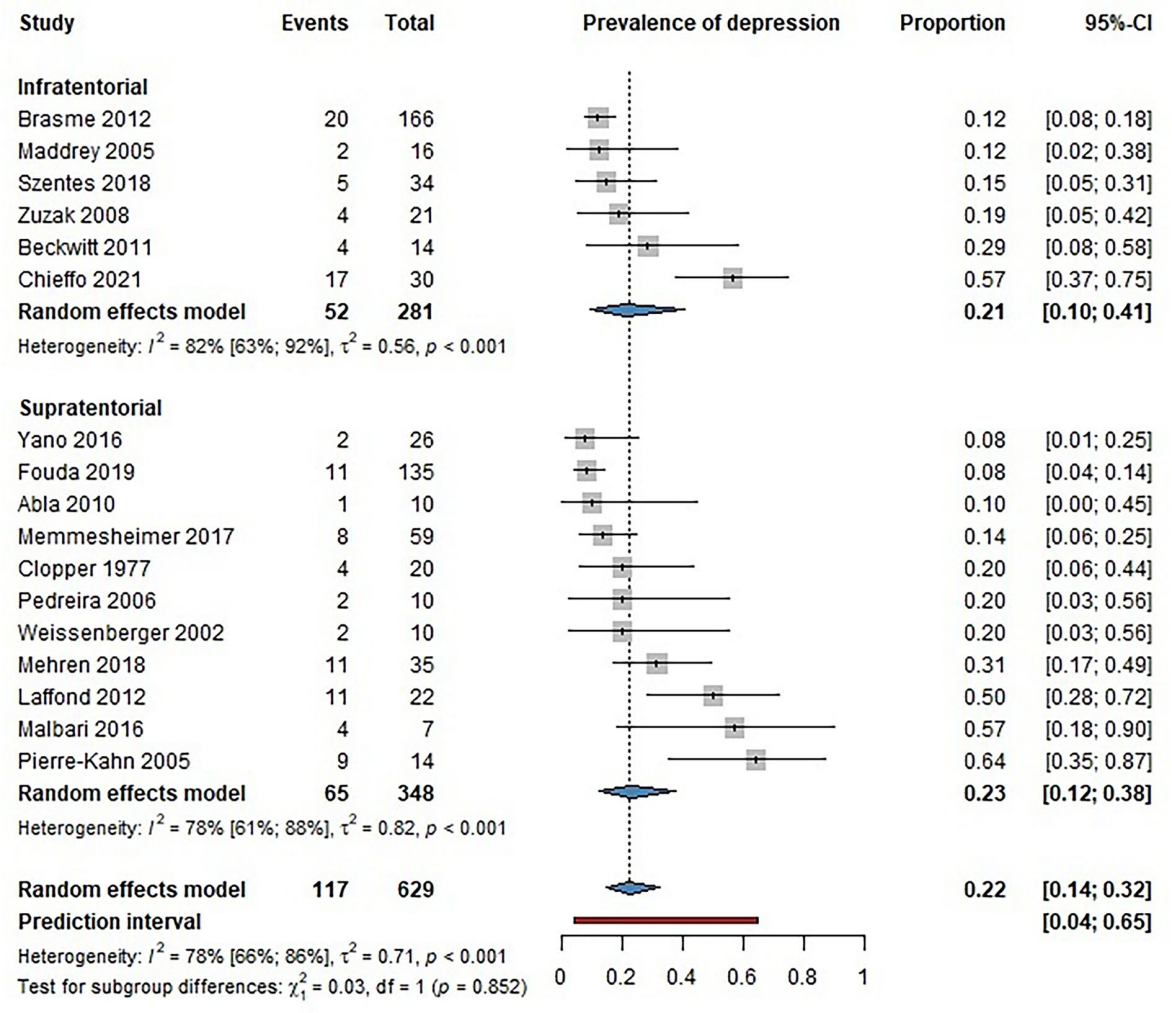
Forest plot showing the prevalence of major depression among childhood brain tumor survivors

The studies included reported major depression diagnosed by professionals among survivors of pediatric brain tumors. The analysis of 17 studies showed no difference in the prevalence of major depression between infratentorial (INF) and supratentorial (SUP) brain tumor survivors [proportions: INF 0.21 (CI 0.10–0.41); SUP 0.23 (CI 0.12–0.38]. The overall prevalence of major depression among survivors of brain tumors was 22% (Fig. 2).

### Brain tumor location and the prevalence of anxiety

**Fig. 3:**
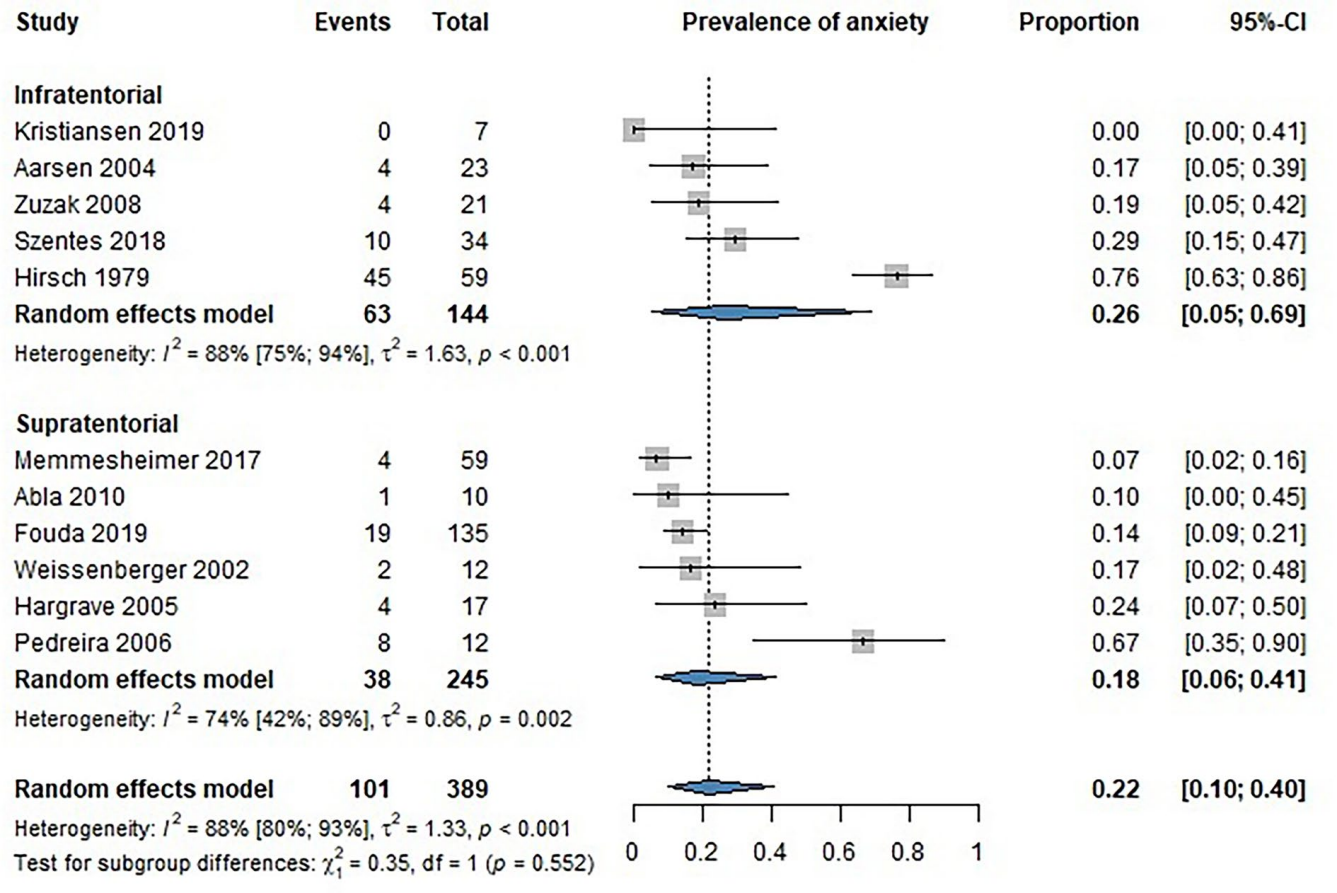
Forest plot showing the prevalence of generalized anxiety disorder among childhood brain tumor survivors

The articles included described anxiety disorders among brain tumor survivors diagnosed by professionals. The pooled analysis of 11 studies showed no statistically significant difference in the prevalence of anxiety between infratentorial (INF) and supratentorial (SUP) brain tumor survivors [proportions: INF 0.26 (CI 0.05–0.69); SUP 0.18 (CI 0.06–0.41]. The overall prevalence of anxiety disorders among survivors of pediatric brain cancer was 22% (Fig. 3).

### Impact of the brain tumor location on the scores of depression assessment instruments

**Fig. 4:**
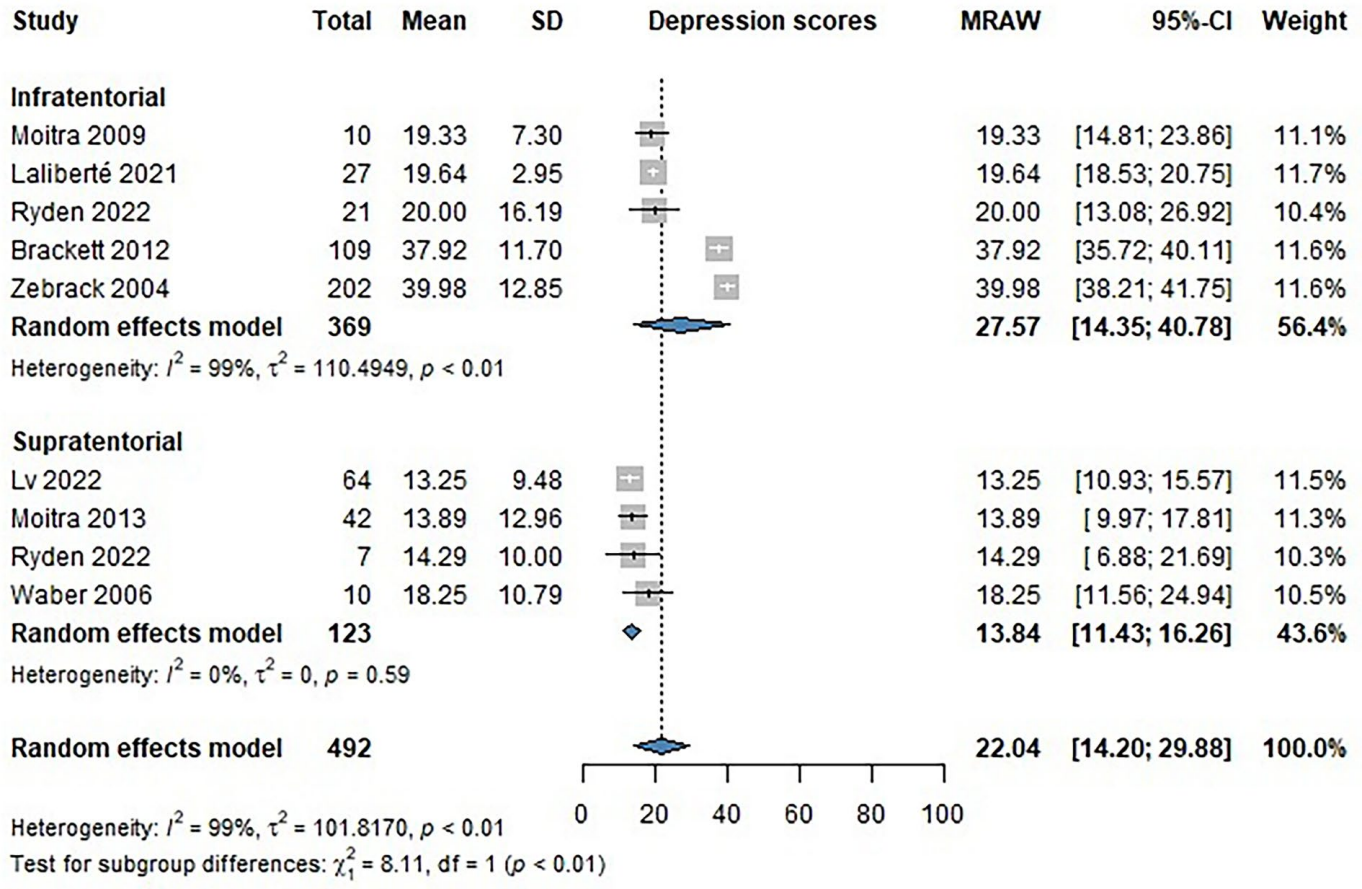
Forest plot showing the mean scores of patients on depression assessment tools (HADS, CDI, CDI-2, BSI-18)

Nine studies reported data using different tools to measure depression in patients with brain tumors and survivors. The assessments included the Children’s Depression Inventory (CDI), Children’s Depression Inventory 2 (CDI-2), Hospital Anxiety and Depression Scale (HADS), and the Brief Syndrome Inventory 18 (BSI-18). The main scores of the different tools were compared on a linear scale [22]. Infratentorial (INF) brain tumor survivors tended to score higher on depression inventories than supratentorial (SUP) brain tumor survivors [INF MRAW: 27.57 (CI 14.35–40.78); SUP MRAW: 13.84 (CI 11.43–16.26), p < 0.01] (Fig. 4).

### Impact of the brain tumor location on the scores of anxiety assessment instruments

**Fig. 5:**
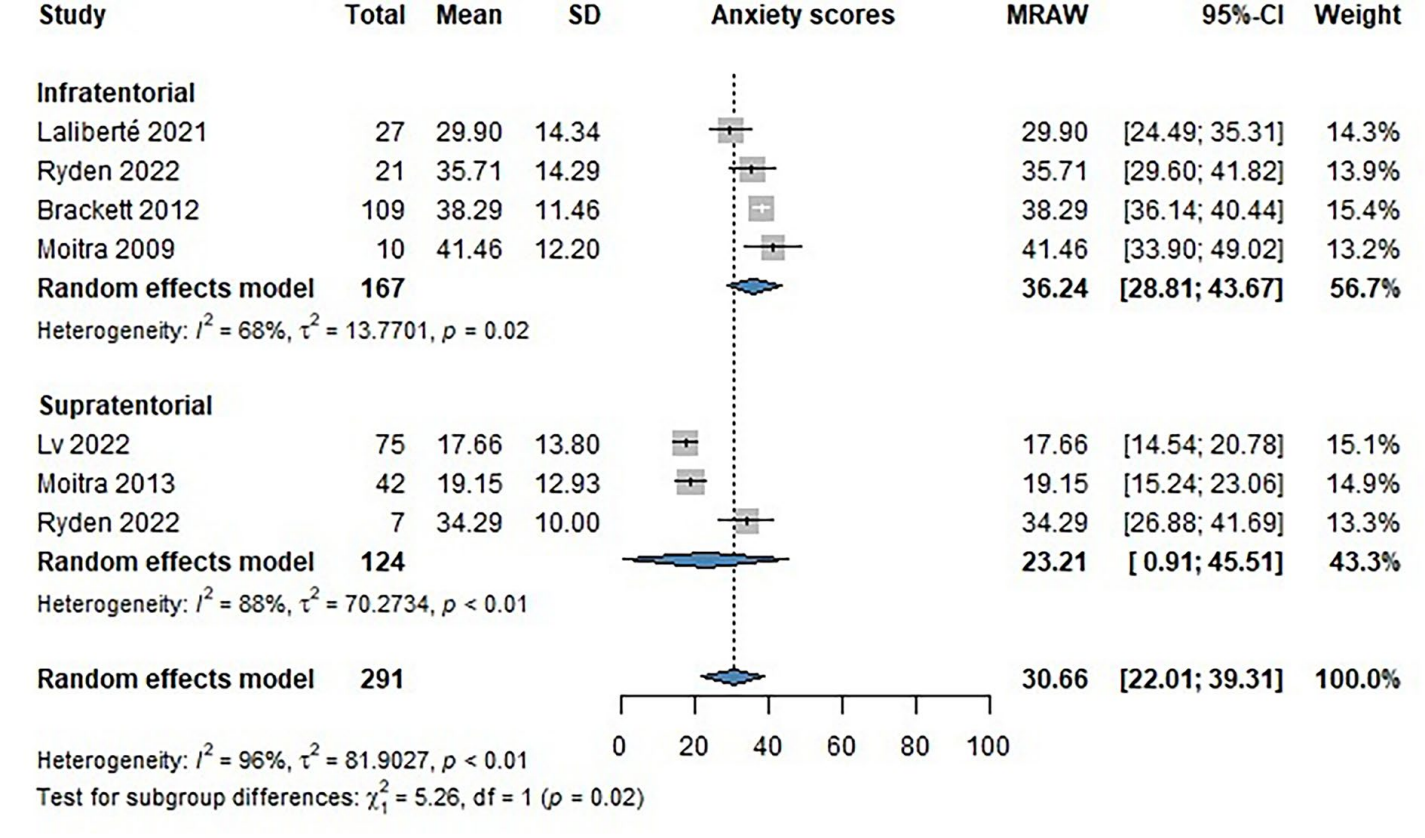
Forest plot showing the mean scores of patients on anxiety assessment tools (HADS, SCARED, BSI-18)

Seven publications provided information on scores using instruments measuring anxiety in pediatric brain tumor survivors. The assessments included the Screen for Child Anxiety Related Disorders (SCARED), Hospital Anxiety and Depression Scale (HADS), and Brief Syndrome Inventory 18 (BSI-18). Infratentorial (INF) brain tumor survivors tended to score higher on depression inventories than supratentorial (SUP) brain tumor survivors. [INF MRAW: 36.24 (CI 28.81–43.67); SUP MRAW: 23.21 (CI 0.91–45.51), p = 0.02] (Fig. 5).

### Impact of the brain tumor location on the scores of the CBCL questionnaire

**Fig. 6:**
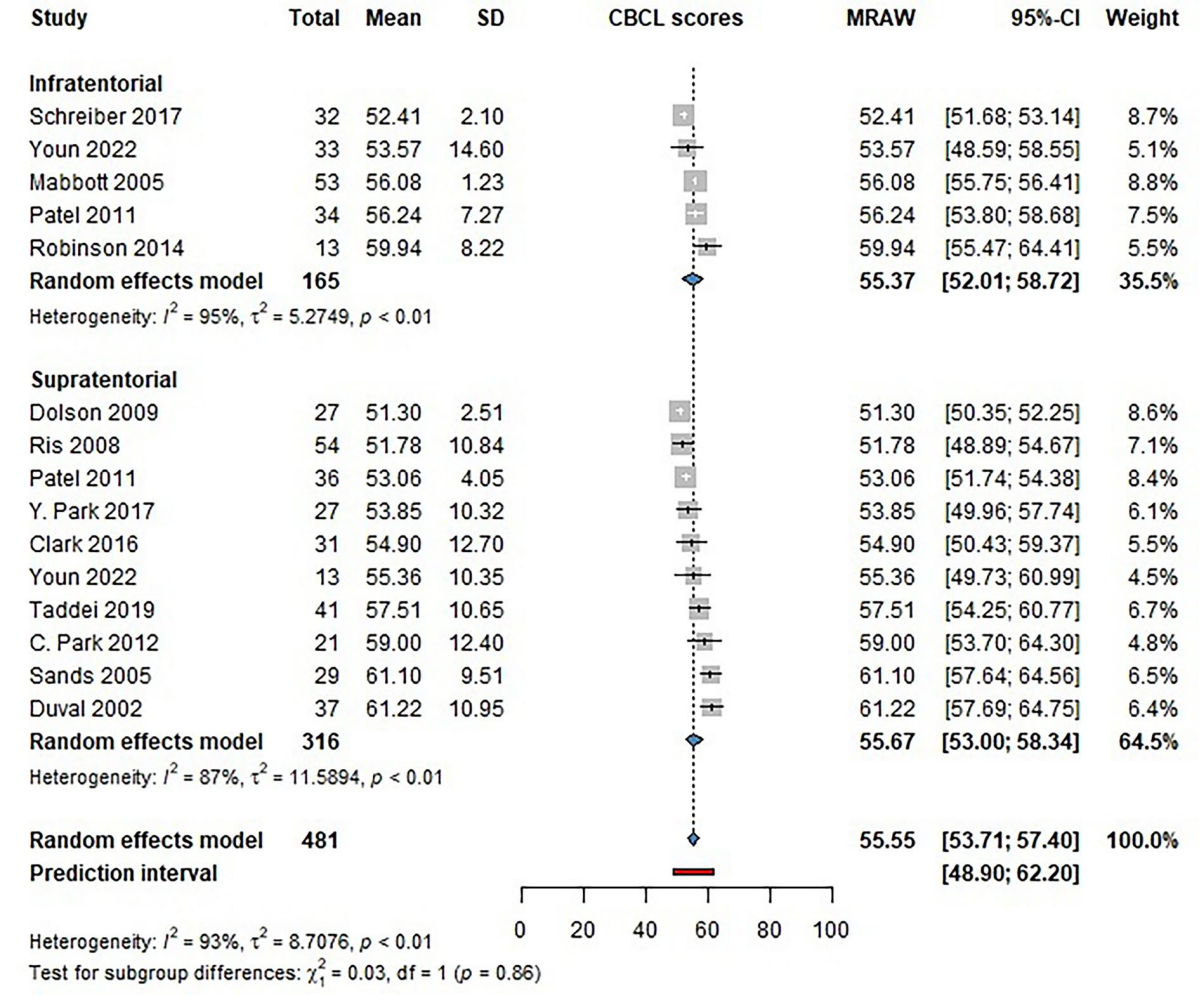
Forest plot showing the T-scores patients achieved on the anxiety/depression domain of the CBCL questionnaire

Fifteen studies reported scores on the Anxiety/Depression part of the Child Behavior Checklist (CBCL) among childhood brain tumor survivors. Studies reported T-scores for this domain of the questionnaire. Our findings showed no significant difference between supratentorial (SUP) and infratentorial (INF) brain tumor survivors [INF score: 55.37 (CI 52.01–58.72); SUP score: 55.67 (CI 53.0–58.34] (Fig. 6).

### Impact of time after treatment on depression and anxiety among brain tumor survivors

As follow-up time is one of the prime factors that may influence the prevalence and rate of affective disorders, we planned to perform a meta-regression based on the different follow-up times used in the studies included. After search and selection, we found that the follow-up times were highly heterogeneous, and patients were evaluated over a wide range of time. Methodically, it would have been inappropriate to use meta-regression for time intervals instead of time points; therefore, we decided to dichotomize the follow-up times and set up two subgroups, with a cut-off time of 48 months and a tolerance of ^±^12 months. After dichotomization, we did not have a sufficient number of articles to perform a sensible subgroup analysis, again we could not draw a valid conclusion regarding the follow-up times (Figure of dichotomization: Additional file 3).

### Risk of bias assessment

The risk of bias is low in 36% of the papers, moderate in 24%, and high in 40%. The significant risk of bias was mainly due to lack of information on confounding factors, and the lack of information on methodology and statistical analyses (Additional file 4).

### Assessment of heterogeneity

Statistical heterogeneity was observed in all the analyses performed. One of the main reasons for the heterogeneity was that different follow-up times were used in each study. Therefore, we performed a subgroup analysis based on the follow-up times, which showed no significant difference between the short- and long-term follow-up of patients, as discussed earlier. Another reason for the perceived heterogeneity is the subjectivity of diagnoses.

## Discussion

The primary objective was to investigate the relationship between tumor location and depression and anxiety.

Childhood brain cancer survivors are at increased risk of psychological and emotional impairments. Previous studies have reported that these impairments are strongly associated with the treatment patients receive; however, our current knowledge about the role and impact of brain tumor location on affective disorders is limited, underlining the necessity of further research in this field [23, 24].

The results of our meta-analysis are consistent with the literature on the prevalence of depression and anxiety among brain tumor survivors. Survivors of pediatric infratentorial tumorsattain worse scores in depression and anxiety assessment tools.

### Childhood brain tumor location and depression

Survivors of pediatric brain tumors are still at a drastically high risk of developing major depression, with about 20% of them suffering from this condition [25], which is about 5 to 10 times higher than that in the healthy population, 3% in children and around 5% in the adult population [26, 27]. Our results correlate with the literature, our meta-analysis found that the overall rate of depression among childhood brain cancer survivors was 22%.

In our analysis, we found no difference in the prevalence of major depression by tumor location. However, the infratentorial group scored significantly higher on the depression assessment tools, indicating that they may be more affected in this field, even if they did not meet the diagnostic threshold.

### Childhood brain tumor location and anxiety

Neighboring depression, anxiety is the most common affective disorder among cancer survivors, affecting approximately 21% of patients [29]. Although the average prevalence of anxiety in healthy children is around 9% [30], the number of survivors with anxiety is worrying. Existing data show that these disorders persist long after active treatment has ended, probably due to fear of cancer recurrence and the late effects of treatment [31]. Our study showed that patients with infratentorial brain tumors achieve higher scores in different anxiety assessment tools, we obserwed significantly worse outcomes in this gorup. This information provides a direction and perspective for further reflection and the need to pay more attention to this subgroup.

### Childhood brain tumor location and depression, anxiety measured by assessment tools

To confirm the diagnosis and follow-up of the patients, professionals use different assessment tools to measure impairments in psychiatric diseases. These questionnaires rate the severity of symptoms related to depression and anxiety, provide impressions and help in early identification of symptoms [32, 33]. Our results showed that patients treated for infratentorial brain tumors tended to score significantly higher for both depression and anxiety. Although our analysis of scores in the CBCL questionnaire showed no differences between the two groups, the overall scores for childhood cancer survivors were above the average observed in the normative sample [34]. These results also suggest that patients treated for brain tumors require closer attention and pursuit by psychologists and professionals in this area.

### Possible reasons behind our findings

There are a number of plausible reasons for the statement that patients treated for infratentorial brain cancer are more likely to develop symptoms of depression and anxiety.

The major symptoms of tumors located in the scala posterior include impairment in motor coordination, lesions of the eye movements, and blinding headache [35]. They can cause patients problems with everyday activities, such as socializing with peers and friends [36].

Another reason for our findings is that, among other treatment modalities, radiation therapy can vary widely based on the location of the tumor. Radiation therapy for infratentorial tumors may require special techniques and careful planning to minimize damage to surrounding normal tissues and avoid functional deficits. In some cases, this may involve using higher doses of radiation therapy to achieve the desired tumor control while sparing nearby critical structures [23]. In our study, we did not investigate the effect of radiation dose on the development of affective disorders, although we know from previous studies that patients with higher radiation doses are more likely to suffer from different adverse effects [37].

Our findings revealed a notable disparity in scores on depression and anxiety assessment tools; however, there was no discernible variance in the diagnosed prevalence of these conditions. There are multiple plausible explanations for this phenomenon. For instance, depression could manifest as subclinical depression, wherein individuals might achieve higher scores on depression assessment tools due to experiencing considerable distress or impairment, despite not meeting the formal criteria for clinical diagnosis. Another contributing factor is the potential influence of cultural norms and personal experiences on how individuals perceive and communicate distress. Additionally, external elements like life events, chronic stressors, and environmental factors could contribute to feelings of sadness and reduced mood, potentially resulting in elevated scores on depression and anxiety assessments. These variables may affect individuals disparately, creating a perceived incongruity between assessment outcomes and diagnostic standards [38, 39].

## Strengths and limitations

To the best of our knowledge, this is the first comprehensive meta-analysis on this topic in the pediatric population, despite its importance. We followed a rigorous methodology and evaluated several psychological measurement tools. We assessed and justified the heterogeneity detected in the analyses. We were able to point out limitations and missing information for further investigation of this topic. Our findings show that the prevalence of depression and anxiety analyzed correlates with the data reported in the literature.

One limitation of our study is that our results were obtained from retrospective cohort and cross-sectional studies. Some of the studies included in the analysis had a rather low number of patients. A further limitation is that comparisons were only made by subgroup analysis, rather than direct comparisons within subgroups. We did not perform subgroup analyses by treatment modalities because of the lack of information and heterogeneity of tumor types. For more precise details regarding the prevalence of affective disorders in the population, meta-regression of the different follow-up times used in different studies should have been performed. As detailed in the first part of the discussion, this analysis could not be performed due to lack of data. An other limitation is that we did not investigate the prevalence of different types of anxiety diagnoses, such as generalized anxiety disorders, panic disorders, social phobia, etc. detailed.

### Implications for practice

On the basis of our findings, regular measurements of the psychological impairment in pediatric brain tumor survivors and close psychological follow-up at regular follow-up checkups (1 month, 3 months, 6 months, 1–5 years after active treatment) are strongly recommended. Professionals should focus more on infratentorial brain tumor survivors during psychological follow-up. Therefore, it is important to amend guidelines following strict rules for the long-term follow-up of childhood brain cancer survivors [40].

### Implications for future research

There is limited information available on this topic; therefore, we suggest further research on the impact of brain tumor location on psychological impairments in children. For a more precise and detailed analysis, we recommend conducting prospective cohort studies with standardized follow-up times and presenting individual patient data on follow-up times in the manuscript (mean, median, SD, quartiles). The protocolized follow-up times used for the clinical evaluation of patients with cancer after treatment were 1 month, 3 months, 6 months, 1 year, 3 years, and 5 years. These follow-up checkups would be useful to measure affective and neurocognitive disorders in these patients. We also recommend the use of harmonized and standardized tools to measure impairments in these fields to facilitate a comparable analysis.

## Conclusion

One in five patients surviving pediatric brain tumor develops major depression and anxiety disorder. Patients who were treated for infratentorial tumors during their childhood are at a higher risk of developing anxiety and also score worse on measures of depression and anxiety impairment. Standardized measurement tools and defined follow-up times are essential for a more accurate analysis of this topic.

### Supplementary Information

**Additional file 1. **List of key search terms that have been used for 5 different databases.

**Additional file 2. **List of articles included in meta-analysis.

**Additional file 3. **Follow-up durations used in different studies.

**Additional file 4. **Risk of Bias Assessment.

## Data Availability

The datasets used in this study can be found either in full-text articles, conference abstracts, personal correspondence, or online databases of clinical research studies included in systematic reviews and meta-analyses.

## Abbreviations

MRAW: Raw mean scores
CI: Confidence interval
HADS: Hospital Anxiety and Depression Scale
CDI: Children’s Depression Inventory
CDI-2: Children’s Depression Inventory 2
BSI-18: Brief Symptom Inventory 18
SCARED: Screen for Child Anxiety Related Disorders
CBCL: Child Behavior Checklist
SUP: Supratentorial
INF: Infratentorial
