# Supplementary material for: Association of tumor location with anxiety and depression in childhood brain cancer survivors: a systematic review and meta-analysis
Source: Child Adolesc Psychiatry Ment Health. 2023 Oct 27;17:124. doi: 10.1186/s13034-023-00665-0 (PMC10612250; doi:10.1186/s13034-023-00665-0)
Supplement: Supplementary file 1 — Additional file 1. List of key search terms that have been used for 5 different databases. [file 13034_2023_665_MOESM1_ESM.pdf]

Article title: Impact of Tumor Location on the Development of Affective Disorders Among Childhood Brain Cancer Survivors - A Systematic Review and Meta-analysis

Journal name: European Child & Adolescent Psychiatry

Author names: Márton Szabados, Erika Kolumbán, Gergely Agócs, Szilvia Kiss-Dala, Marie Anne Engh, Márk Hernádfői, Kata Takács, Eszter Tuboly, Andrea Párniczky, Péter Hegyi, Miklós Garami

Corresponding author:
Miklós Garami, MD, MSc, PhD
Corresponding author
Pediatric Center, Semmelweis University, Budapest, Hungary
7-9 Tűzoltó Str., 1094 Budapest, Hungary
mobile: +36 (20) 825-9253
phone: +36 (1) 215-1380
email: garami.miklos@semmelweis.hu
ORCID: 0000-0003-4298-2746

Pediatric: (pediatric* OR paediatric* OR adolescent OR adolescence OR child* OR "young adult" OR "young adults" OR kids OR kid OR youth OR juvenile OR infant* OR infancy OR preschooler* OR teen OR teens OR teenager*) **AND**

Brain cancer: (“posterior fossa syndrome” OR astrocytoma OR glioblastoma OR DIPG OR “glioma” OR “HGG” OR “LGG” OR “ATRT” OR “PNET” OR medulloblastoma OR dysgerminoma OR oligodendroglioma OR xanthoastrocytoma OR astroblastoma OR ganglioglioma OR gangliocytoma OR "Lhermitte-Duclos disease" OR "neurocytoma" OR "cerebellar liponeurocytoma" OR ependymoma OR subependymoma OR "choroid plexus papilloma” OR pineocytoma OR pineoblastoma OR schwannoma OR neurofibroma OR perineurioma OR paraganglioma OR meningeoma OR "CNS hemangioma" OR "CNS vascular malformation" OR "meningeal melanocytosis" OR "meningeal melanomatosis" OR "meningeal melanocytoma" OR germinoma OR choriocarcinoma OR craniopharyngeoma OR pituicytoma OR oncocytoma OR "pituitary adenoma" OR "PitNET" OR "pituitary blastoma" OR "brain cancer" OR "brain cancers" OR “central nervous system tumor” OR “central nervous system tumour” OR “brain malignoma”) **AND**

Localization: (localization OR localiz* OR "frontal lobe" OR frontal OR "temporal lobe" OR temporal OR "cerebellum" OR cerebellar* OR parietal OR "parietal lobe" OR "brain stem" OR "occipital lobe" OR occipital OR "supratentorial" OR "infratentorial" OR "thalamus" OR "hypothalamus" OR "pineal gland" OR pituitary OR amygdala OR "corpus callosum") **AND**

Affective disorders: ("mental health" OR mental health OR psychology OR psychologic* OR "mental issues" OR mental issue* OR mood* OR emotion* OR affective disorders OR affective OR "mental disorder" OR mental disord* OR depressi* OR "major depression" OR "anxiety" OR anxie* OR "unipolar depression" OR "bipolar depression" OR bipolar* OR dysthymia OR cyclothymia OR CDI OR CBCL OR SCARED OR BDI OR CDS)

Search key:

(pediatric* OR paediatric* OR adolescent OR adolescence OR child* OR "young adult" OR "young adults" OR kids OR kid OR youth OR juvenile OR infant* OR infancy OR preschooler* OR teen OR teens OR teenager*) AND (“posterior fossa syndrome” OR astrocytoma OR glioblastoma OR DIPG OR “glioma” OR “HGG” OR “LGG” OR “ATRT” OR “PNET” OR medulloblastoma OR dysgerminoma OR oligodendroglioma OR xanthoastrocytoma OR astroblastoma OR ganglioglioma OR gangliocytoma OR "Lhermitte-Duclos disease" OR "neurocytoma" OR "cerebellar liponeurocytoma" OR ependymoma OR subependymoma OR "choroid plexus papilloma” OR pineocytoma OR pineoblastoma OR schwannoma OR neurofibroma OR perineurioma OR paraganglioma OR meningeoma OR "CNS hemangioma" OR "CNS vascular malformation" OR "meningeal melanocytosis" OR "meningeal melanomatosis" OR "meningeal melanocytoma" OR germinoma OR choriocarcinoma OR craniopharyngeoma OR pituicytoma OR oncocytoma OR "pituitary adenoma" OR "PitNET" OR "pituitary blastoma" OR "brain cancer" OR "brain cancers" OR “central nervous system tumor” OR “central nervous system tumour” OR “brain malignoma”) AND (localization OR localiz* OR "frontal lobe" OR frontal OR "temporal lobe" OR temporal OR "cerebellum" OR cerebellar* OR parietal OR "parietal lobe" OR "brain stem" OR "occipital lobe" OR occipital OR "supratentorial" OR "infratentorial" OR "thalamus" OR "hypothalamus" OR "pineal gland" OR pituitary OR amygdala OR "corpus callosum") AND ("mental health" OR mental health OR psychology OR psychologic* OR "mental issues" OR mental issue* OR mood* OR emotion* OR affective disorders OR affective OR "mental disorder" OR mental disord* OR depressi* OR "major depression" OR "anxiety" OR anxie* OR "unipolar depression" OR "bipolar depression" OR bipolar* OR dysthymia OR cyclothymia OR CDI OR CBCL OR SCARED OR BDI OR CDS)
