# Supplementary material for: Association of tumor location with anxiety and depression in childhood brain cancer survivors: a systematic review and meta-analysis
Source: Child Adolesc Psychiatry Ment Health. 2023 Oct 27;17:124. doi: 10.1186/s13034-023-00665-0 (PMC10612250; doi:10.1186/s13034-023-00665-0)
Supplement: Supplementary file 2 — Additional file 2. List of articles included in meta-analysis. [file 13034_2023_665_MOESM2_ESM.pdf]

Article title: Impact of Tumor Location on the Development of Affective Disorders Among Childhood Brain Cancer Survivors - A Systematic Review and Meta-analysis

Journal name: European Child & Adolescent Psychiatry

Author names: Márton Szabados, Erika Kolumbán, Gergely Agócs, Szilvia Kiss-Dala, Marie Anne Engh, Márk Hernádfői, Kata Takács, Eszter Tuboly, Andrea Párniczky, Péter Hegyi, Miklós Garami

Corresponding author:
Miklós Garami, MD, MSc, PhD
Corresponding author
Pediatric Center, Semmelweis University, Budapest, Hungary
7-9 Tűzoltó Str., 1094 Budapest, Hungary
mobile: +36 (20) 825-9253
phone: +36 (1) 215-1380
email: garami.miklos@semmelweis.hu
ORCID: 0000-0003-4298-2746

	Author, year	Country	N°of patients	Major localization	Type of affective disorder	Assessed by*
1	Abla, 2010	United States	10	Supratentorial	Major depressive disorder, Generalized anxiety disorder	Professional
2	Beckwitt, 2011	United States	14	Infratentorial	Major depressive disorder	Professional
3	Brasme, 2012	France	166	Infratentorial	Major depressive disorder	Professional
4	Fouda, 2019	United States	135	Supratentorial	Major depressive disorder, Generalized anxiety disorder	Professional
5	Maddrey, 2005	United States	16	Infratentorial	Major depressive disorder	Professional
6	Malbari, 2016	United States	7	Supratentorial	Major depressive disorder	Professional
7	Zuzak, 2008	Schwitzerland	21	Infratentorial	Major depressive disorder, Generalized anxiety disorder	Professional
8	Yano, 2016	Japan	26	Supratentorial	Major depressive disorder	Professional
9	Chieffo, 2021	Italy	30	Infratentorial	Major depressive disorder	Professional
10	Clopper, 1977	United States	20	Supratentorial	Major depressive disorder	Professional
11	Laffond, 2012	France	22	Supratentorial	Major depressive disorder	Professional
12	Mehren, 2018	Germany	35	Supratentorial	Major depressive disorder	Professional
13	Memmesheimer, 2017	Germany	59	Supratentorial	Major depressive disorder, Generalized anxiety disorder	Professional
14	Pedreira, 2006	Australia	10	Supratentorial	Major depressive disorder, Generalized anxiety disorder	Professional
15	Szentes, 2018	Hungary	34	Infratentorial	Major depressive disorder, Generalized anxiety disorder	Professional
16	Weissenberger, 2002	United States	12	Supratentorial	Major depressive disorder, Generalized anxiety disorder	Professional
17	Pierre-Kahn, 2005	France	14	Supratentorial	Major depressive disorder	Professional
18	Hargrave, 2005	Canada	17	Supratentorial	Generalized anxiety disorder	Professional
19	Aarsen, 2004	Netherlands	23	Infratentorial	Generalized anxiety disorder	Professional
20	Hirsch, 1979	France	59	Infratentorial	Generalized anxiety disorder	Professional
21	Kristiansen, 2019	Sweden	7	Infratentorial	Generalized anxiety disorder	Professional
22	Sands, 2005	United States	29	Supratentorial	Anxiety/Depression	CBCL
23	Duval, 2002	Canada	37	Supratentorial	Anxiety/Depression	CBCL
24	Y. Park, 2017	Republic of Korea	27	Supratentorial	Anxiety/Depression	CBCL
25	Patel, 2011	United States	70	Supratentorial and Infratentorial	Anxiety/Depression	CBCL
26	C. Park, 2012	United States	21	Supratentorial	Anxiety/Depression	CBCL
27	Mabbott, 2005	Canada	53	Infratentorial	Anxiety/Depression	CBCL
28	Dolson, 2009	United States	27	Supratentorial	Anxiety/Depression	CBCL
29	Ris, 2008	United States	54	Supratentorial	Anxiety/Depression	CBCL
30	Robinson, 2014	United States	13	Infratentorial	Anxiety/Depression	CBCL
31	Youn, 2022	Republic of Korea	46	Supratentorial and infratentorial	Anxiety/Depression	CBCL
32	Taddei, 2019	Italy	41	Supratentorial	Anxiety/Depression	CBCL
33	Schreiber, 2017	United States	32	Infratentorial	Anxiety/Depression	CBCL
34	Clark, 2016	United States	31	Supratentorial	Anxiety/Depression	CBCL
35	Ryden, 2022	Sweden	28	Supratentorial and infratentorial	Depression, anxiety	HADS
36	Lv, 2022	China	64	Supratentorial	Depression, anxiety	CDI, SCARED
37	Moitra, 2009	United States	10	Infratentorial	Depression, anxiety	CDI, SCARED
38	Moitra, 2013	United States	42	Supratentorial	Depression, anxiety	CDI, SCARED
39	Laliberté, 2021	Canada	27	Infratentorial	Depression, anxiety	CDI-2, SCARED
40	Brackett, 2012	United States	109	Infratentorial	Depression, anxiety	BSI-18
41	Zebrack, 2004	United States	202	Infratentorial	Depression	BSI-18
42	Waber, 2006	United States	10	Supratentorial	Depression	BDI

*We included studies in our meta-analyses that showed clinical diagnosis of depression or anxiety
or had data about the scores on different assessment tools (CBCL, CDI, CDI-2, BDI, BSI-18, HADS, SCARED)
