# Supplementary material for: Association of tumor location with anxiety and depression in childhood brain cancer survivors: a systematic review and meta-analysis
Source: Child Adolesc Psychiatry Ment Health. 2023 Oct 27;17:124. doi: 10.1186/s13034-023-00665-0 (PMC10612250; doi:10.1186/s13034-023-00665-0)
Supplement: Supplementary file 3 — Additional file 3. Follow-up durations used in different studies. [file 13034_2023_665_MOESM3_ESM.pdf]

Article title: Impact of Tumor Location on the Development of Affective Disorders Among Childhood Brain Cancer Survivors - A Systematic Review and Meta-analysis

Journal name: European Child & Adolescent Psychiatry

Author names: Márton Szabados, Erika Kolumbán, Gergely Agócs, Szilvia Kiss-Dala, Marie Anne Engh, Márk Hernádfői, Kata Takács, Eszter Tuboly, Andrea Párniczky, Péter Hegyi, Miklós Garami

Corresponding author:

Miklós Garami, MD, MSc, PhD

Corresponding author

Pediatric Center, Semmelweis University, Budapest, Hungary

7-9 Tűzoltó Str., 1094 Budapest, Hungary

mobile: +36 (20) 825-9253

phone: +36 (1) 215-1380

email: garami.miklos@semmelweis.hu

ORCID: 0000-0003-4298-2746

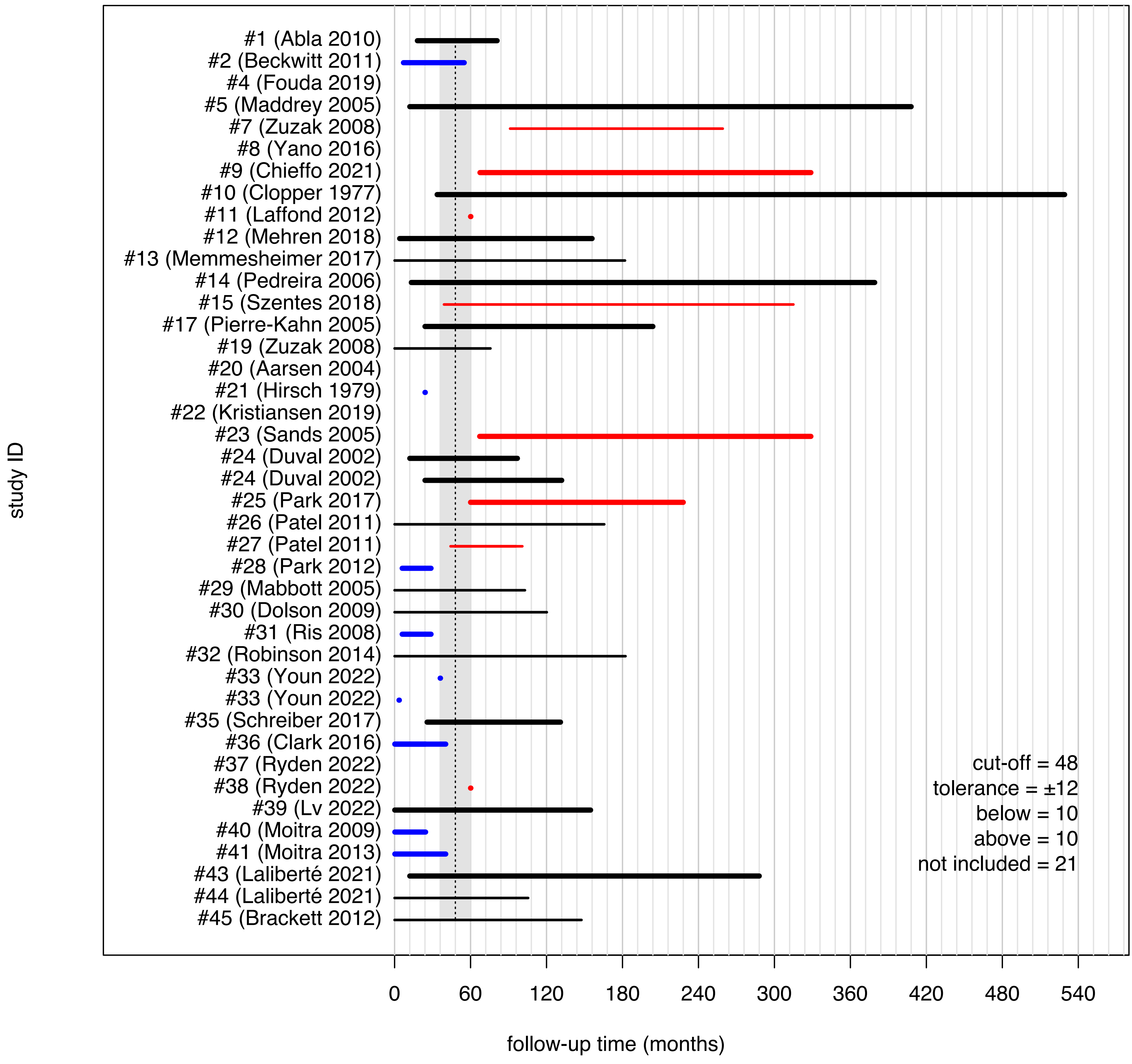
#1 (Abla 2010)
#2 (Beckwitt 2011)
#4 (Fouda 2019)
#5 (Maddrey 2005)
#7 (Zuzak 2008)
#8 (Yano 2016)
#9 (Chieffo 2021)
#10 (Clopper 1977)
#11 (Laffond 2012)
#12 (Mehren 2018)
#13 (Memmesheimer 2017)
#14 (Pedreira 2006)
#15 (Szentes 2018)
#17 (Pierre-Kahn 2005)
#19 (Zuzak 2008)
#20 (Aarsen 2004)
#21 (Hirsch 1979)
#22 (Kristiansen 2019)
#23 (Sands 2005)
#24 (Duval 2002)
#24 (Duval 2002)
#25 (Park 2017)
#26 (Patel 2011)
#27 (Patel 2011)
#28 (Park 2012)
#29 (Mabbott 2005)
#30 (Dolson 2009)
#31 (Ris 2008)
#32 (Robinson 2014)
#33 (Youn 2022)
#33 (Youn 2022)
#35 (Schreiber 2017)
#36 (Clark 2016)
#37 (Ryden 2022)
#38 (Ryden 2022)
#39 (Lv 2022)
#40 (Moitra 2009)
#41 (Moitra 2013)
#43 (Laliberté 2021)
#44 (Laliberté 2021)
#45 (Brackett 2012)
study ID
cut-off = 48
tolerance = ±12
below = 10
above = 10
not included = 21
0
60
120
180
240
300
360
420
480
540
follow-up time (months)

Additional file 3

Follow-up durations used in different studies.
The time intervals are given in months.
We performed a dichotomization, with the cut-off time of 48 months and 12 months of tolerance.
We chose the cut-off value based on both clinical and methodological decision.
Follow-up under- 48+12 months: short-term follow-up (blue lines) Follow-up above months: long-term follow-up (red lines).
Studies crossing the cut-off values were not included in the analyzis.
After performing the dichotomization, we still did not have enough studies to have a clear conclusion about the impact of follow-up times on the affective disorders.
