# Supplementary material for: Association of tumor location with anxiety and depression in childhood brain cancer survivors: a systematic review and meta-analysis
Source: Child Adolesc Psychiatry Ment Health. 2023 Oct 27;17:124. doi: 10.1186/s13034-023-00665-0 (PMC10612250; doi:10.1186/s13034-023-00665-0)
Supplement: Supplementary file 4 — Additional file 4. Risk of Bias Assessment. [file 13034_2023_665_MOESM4_ESM.pdf]

Article title: Impact of Tumor Location on the Development of Affective Disorders Among Childhood Brain Cancer Survivors - A Systematic Review and Meta-analysis

Journal name: European Child & Adolescent Psychiatry

Author names: Márton Szabados, Erika Kolumbán, Gergely Agócs, Szilvia Kiss-Dala, Marie Anne Engh, Márk Hernádfői, Kata Takács, Eszter Tuboly, Andrea Párniczky, Péter Hegyi, Miklós Garami

Corresponding author:
Miklós Garami, MD, MSc, PhD
Corresponding author
Pediatric Center, Semmelweis University, Budapest, Hungary
7-9 Tűzoltó Str., 1094 Budapest, Hungary
mobile: +36 (20) 825-9253
phone: +36 (1) 215-1380
email: garami.miklos@semmelweis.hu
ORCID: 0000-0003-4298-2746

Risk of bias domains

Study	D1	D2	D3	D4	D5	D6	Overall
Aarsen 2004	+	?	+	+	-	+	-
Abla 2010	+	?	+	+	-	+	-
Beckwitt 2012	-	?	+	-	X	X	X
Brackett 2012	+	?	+	+	+	+	+
Brasme 2012	-	?	+	X	+	+	X
Chieffo 2021	+	+	+	+	-	+	+
Clark 2016	+	?	+	+	X	+	X
Clopper 1977	-	?	+	+	-	X	X
Dolson 2009	+	?	+	-	-	+	-
Duval 2002	-	?	+	+	+	+	+
Fouda 2020	+	?	+	+	+	X	X
Hargrave 2006	-	?	+	X	X	X	X
Hirsch 1979	+	?	+	-	+	X	X
Kristiansen 2019	-	+	+	+	-	+	-
Laffond 2012	+	?	+	+	+	+	+
Laliberté 2021	+	?	+	+	+	+	+
Lv 2022	+	?	+	+	+	+	+
Mabbott 2005	-	?	+	-	-	+	X
Maddrey 2005	+	?	+	+	X	+	X
Malbari 2016	-	?	+	X	X	X	X
Mehren 2018	+	+	+	+	-	+	-
Memmesheimer 2017	+	-	+	+	-	+	-
Moitra 2009	+	?	+	+	+	+	+
Moitra 2013	+	?	+	+	+	+	+
Park 2013	+	?	+	X	-	+	X
Park 2017	+	-	+	+	+	+	-
Patel 2011	+	?	+	+	+	+	+
Pedreira 2006	+	?	+	+	X	+	X
Pierre-Kahn 2005	+	+	+	+	-	X	X
Ris 2008	+	?	+	+	-	+	-
Robinson 2015	+	+	+	+	+	+	+
Rydén 2022	+	?	+	+	+	+	+
Sands 2005	+	?	+	+	-	+	-
Schreiber 2017	+	+	+	+	+	+	+
Szentes 2018	+	+	+	+	+	+	+
Taddei 2019	-	?	+	X	-	+	X
Waber 2006	+	+	+	+	-	+	-
Weissenberger 2001	+	+	+	X	X	+	X
Yano 2016	+	?	+	+	+	+	+
Youn 2022	+	+	+	+	+	+	+
Zebrack 2004	+	?	+	X	+	+	X
Zuzak 2008	+	?	+	+	X	X	X

Domains:
D1: Bias due to participation.
D2: Bias due to attrition.
D3: Bias due to prognostic factor measurement.
D4: Bias due to outcome measurement.
D5: Bias due to confounding.
D6: Bias in statistical analysis and reporting.

Judgement
X High
- Moderate
+ Low
? No information
